# Bibliography of studies on hybrid zones of the common shrew chromosome races distributed in Russia

**DOI:** 10.3897/CompCytogen.v7i4.6159

**Published:** 2013-11-21

**Authors:** Rena S. Nadjafova

**Affiliations:** 1 A.N. Severtsov Institute of Ecology and Evolution, Russian Academy of Sciences 33 Leninsky pr., 119071 Moscow, Russia

**Keywords:** Chromosome races, Hybrid zones, Robertsonian variation, *Sorex araneus*

## Abstract

The common shrew, *Sorex araneus* Linnaeus, 1758, has become a model species for cytogenetic and evolutionary studies after discovery of extraordinary Robertsonian polymorphism at the within-species level. Development of differential staining techniques (Q-, R-and G-banding) made it possible to identify the chromosomal arms and their combination in racial karyotypes. Entering into contact with each other, the chromosomal races might form hybrid zones which represent a great interest for understanding of the process of speciation. Until recently all known hybrid zones of *S. araneus* were localized in Western Europe and only one was identified in Siberia (Russia) between Novosibirsk and Tomsk races (Aniskin and Lukianova 1989, Searle and Wójcik 1998, Polyakov et al. 2011). However, a rapidly growing number of reports on discovery of interracial hybrid zones of *Sorex araneus* in the European part of Russia and neighboring territories appeared lately. The aim of the present work is to compile the bibliography of all studies covering this topic regardless of the original language and the publishing source which hopefully could make research data more accessible to international scientists. It could also be a productive way to save current history of *Sorex araneus* researches in full context of the ISACC (International *Sorex araneus* Cytogenetics Committee) heritage (Searle et al. 2007, Zima 2008).

## Introduction

The common shrew, *Sorex araneus* Linnaeus, 1758, displays exceptional variability of karyotype derived from intraspecific chromosome rearrangements of the Robertsonian type. Metacentric pairs of *Sorex araneus* are formed by fusion of originally acrocentric chromosomes at their centromeres in different combinations of arms. As a result, the chromosomes number (2n) varies from 20 to 33, the odd number is due to the presence of karyotype of the Robertsonian heterozygote with one metacentric and two acrocentrics, instead of two homozygous metacentrics or four acrocentrics. At the same time the fundamental number of chromosome arms (FN) remains unchanged and is equal to 40. As far as this process takes place within populations, we could talk about Robertsonian polymorphism which occurs in the vast range of *Sorex araneus* species.

After the pioneer analysis in Western Europe in the 1950s and 1960s, the studies of Robertsonian polymorphism in *Sorex araneus* populations started in Russia, widening the area of cytogenetic investigations to include European and Asian parts of the former USSR (Orlov 1974). The observed variations in chromosome arm lengths led to conclusion that Robertsonian fusions might involve different arms in different populations, which resulted in widely varying non-homologous metacentrics (Orlov and Kozlovsky 1969, Ford and Hamerton 1970, Hausser et al. 1985).

Introduction of new methods of chromosome identification (Q-, R- and G-banding) improved the karyotype definition and increased the interest in the common shrew chromosome evolution. The International *Sorex araneus* Cytogenetics Committee, ISACC was founded at Oxford University in 1987 and until recently international meetings were held every 3 years. The results of its activity were summarized in 2007 by Searle et al. Based on chromosome specific G-banding patterns, Searle et al. (1991) established the standard nomenclature for chromosomes of *Sorex araneus*. Later rules for differentiation of the intrapopulation variants (polymorphism) from the interpopulation ones (polytypy) as well as from individual karyotype forms were developed (Hausser et al. 1994). Chromosome identification made it possible to describe the chromosomal races of *Sorex araneus* (Halkka et al. 1974, 1987). Results of karyological studies over the full species range were successively summarized first by Zima et al. (1996) and then by Wójcik et al. (2003). In Russia G-banded chromosomes of the common shrew were first described for a Siberian (Novosibirsk) population by Král and Radjabli in 1974. Results of further studies of high resolution G-banding and chromosome painting of race Novosibirsk represented the species in the international “Atlas of Mammalian Chromosomes” (2006) and in comprehensive comparative studies of *Sorex* (Biltueva et al. 2011).This race was also used for DAPI karyotyping of the common shrew (Minina et al. 2007).

Currently, no less than 72 chromosomal races are recognized in total (White et al. 2010). The number of Russian chromosomal races has already reached 25 (Orlov et al. 1996, 2007, Bulatova et al. 2000, Shchipanov et al. 2009, Pavlova 2010). Only four of these races are common for Russia and some neighboring areas. They include the following: 1) the Neroosa race which spreads over the southern regions of Russia and Ukraine; 2) the West Dvina race which can be found in Russia – Belarus neighboring regions; 3) the Goldap race which inhabits the Baltic coast area of Poland and Kaliningrad region of western Russia; 4) the Ilomantsi race which occurs in the bordering areas of north-western Russia (Karelia) and Finland (Orlov et al. 1996, 2007, Bulatova et al. 2000, Shchipanov et al. 2009, Borisov et al. 2009a). As anticipated, regular studies of distribution of different races resulted in discoveries of interracial zones of contact in Russia (Shchipanov et al. 2009, Orlov et al. 2012, Pavlova 2013, Shchipanov and Pavlova 2013) and neighboring territories (Borisov et al. 2010, 2013). Due to ISACC activity, research that involves detection of the hybrid zones, as well as discovery and description of the chromosome races continues on a regular basis.

The first case of *Sorex araneus* interracial hybridization in Russia was presented by Aniskin and Lukianova (1989) for Tomsk and Novosibirsk races in Western Siberia. This hybrid zone is characterized by the high number of the chromosome arm combinations and remains one of the most complex and best studied *Sorex araneus* hybrid zones (Searle and Wójcik 1998, Polyakov et al. 2011). The hybrids here form a complex meiotic configuration, a long chain of 9 monobrachially homologous acrocentrics and metacentrics. Presumably, chromosome incompatibility proved by meiosis data may induce infertility in hybrids which, in turn, could contribute to promotion of the selection for assortative mating (Searle and Wójcik 1998). Given that racial karyotypes of *Sorex araneus* as a rule differ by 1–5 variable metacentrics, the hybrids should produce rings or chains of different numbers and length in meiosis. Thus, the simplest heterozygotes form the chain of three, CIII, or ring of four, RIV. The most complex heterozygote was registered in Moscow and Seliger races hybrids in European Russia, and represents the chain of eleven, CXI (Bulatova et al. 2007). As far as the meiotic complications may lead to reduced hybrid reproductive fitness, the incompatibility is to be considered as the first stage in reproductive isolation. There are indications that the Robertsonian rearrangements do not interrupt the existent gene flow in hybrid zones and could not promote speciation in *Sorex araneus*. Instead, races might be merely remnants of past allopatric differentiation followed by the loss of secondary contact (Horn et al. 2012, Polly et al. 2013), presenting in particular astonishing racial ‘patchwork’.

As has been shown in a variety of recent studies, the number and diversity of the chromosome rearrangements along with the relative variety of hybrid zone types represent a great opportunity both for understanding of the aftereffects and possible connections of chromosome mutations with the morphological, ecological and genetic differentiation in wild populations of common shrews (see Bibliographic list). It seems quite appropriate to recall the forecast made the British cytogeneticists CE Ford and JL Hamerton in 1970 (p. 235): “… shrews displayed multiple patterns of chromosome variation predicting the problems essential for the interpretation of species evolution. Information about hybrid meiosis would be of outstanding value and studies of pregnant females and their embryos from polymorphic populations could give important information about the breeding system and relative fertility. At a more modest level there remain many parts of Europe from which simple identification of the karyotype in samples from the local population could at least help to fill in the still rather fragmentary distribution map of Races A and B and might reveal further unsuspected chromosome variation”. Till now only the second part of this task has been mostly accomplished, while our knowledge of the influence of chromosome rearrangements on cells, specimen and species is still too fragmentary.

The first tribute to the bibliography on the *Sorex araneus* cytogenetic model was paid by Prof. Jan Zima at the 8th ISACC meeting (2008). To support his idea, we compiled the bibliographical list which includes majority if not all of currently available papers devoted to interracial hybrid zones of *Sorex araneus* in Russia. The Bibliographic list presented here includes 43 full papers published in national and international scientific editions within the last 40 years. As it shown by the published data, hybrid karyotypes and true hybrid zones were reported for at least 14 out of 25 chromosome races (which are indexed below) of the common shrew that inhabit Russia. This index includes the names of the races and their standard abbreviations, karyotypic diagnosis and F1 hybrids meiotic formula followed by the reference number of the relevant papers from our Bibliographic list.

## Bibliographic list *

*Papers from the Bibliographic list referred to in the Introduction and not included in the final References are marked with asterisks.

1. *AniskinVMLukianovaIV (1989) A new chromosome race and hybridization zone analysis of two *Sorex araneus* (Insectivora, Soricidae) karyoforms.Doklady Academii Nauk SSSR309: 1260–1262
[English Translation (1990): Doklady Biological Sciences (Proceedings of the Academy of Sciences of the USSR) 309: 826–829.]2.BannikovaAABulatovaNSKramerovDA (2006) Molecular variability in the common shrew *Sorex araneus* L. from European Russia and Siberia inferred from the length polymorphism of DNA regions flanked by short interspersed elements (Inter-SINE PCR) and the relationships between the Moscow and Seliger chromosome races.Genetika42: 737–747
[English Translation: Russian Journal of Genetics 42: 595–604.]10.1134/S1022795406060020168717773.BorisovYMCherepanovaEVOrlovVN (2010) A wide hybrid zone of chromosomal races of the common shrew, *Sorex araneus* Linnaeus, 1758 (Mammalia), between the Dnieper and Berezina rivers (Belarus).Comparative Cytogenetics4: 195-201.10.3897/compcytogen.v4i2.434.BorisovYMKovalevaAAIrkhinSYOrlovVN (2009) Zones of contact and joint occurrence of three chromosomal races of the common shrew *Sorex araneus* L. (Mammalia) in the southern Valdai Hills.Doklady Academii Nauk428: 275–277
[English Translation: Doklady Biological Sciences 428: 437–439.]10.1134/s0012496609050135199947845. *BorisovYMKovalevaAASpringerAMCherepanovaEVKashtal’ianAPOrlovVN (2009a) Hybrid origin of karyotypic variation in the common shrew, *Sorex araneus* (Mammalia), from the Dnieper river basin.Doklady Academii Nauk429: 561–564
[English translation: Doklady Biological Sciences 429: 531–534.]10.1134/s0012496609060143201700656.BorisovYMKozlovskyAIBalakirevAEDemidovaTBIrchinSYuMaliginVMOkulovaNMPotapovSGShchipanovANOrlovVN (2008) Zones of contact of the chromosome races of the common shrew *Sorex araneus* L. (Insectivora, Mammalia) at the extremes of the Veps stage of the Valdai ice sheet.Siberian Journal of Ecology15: 763-771
[In Russian]7.BorisovYMKryshchukIACherepanovaEVGajduchenkoHSOrlovVN (2013) Chromosomal polymorphism of populations of the common shrew, *Sorex araneus* L., in Belarus.Acta Theriologica.10.1007/s13364-013-0160-y8. *BulatovaNShJonesRMWhiteTAShchipanovNAPavlovaSVSearleJB (2011) Natural hybridization between extremely divergent chromosomal races of the common shrew (*Sorex araneus*, Soricidae, Soricomorpha): hybrid zone in European Russia.Journal of Evolutionary Biology24: 573-586.10.1111/j.1420-9101.2010.02191.x2115900410.1111/j.1420-9101.2010.02191.x9.BulatovaNPavlovaS (2007) The chromosome race in the epicenter of hybrid zones.The Herald of Vavilov Society for geneticists and breeding scientists11: 432–435
http://www.bionet.nsc.ru/vogis/pict_pdf/2007/t11_2/vogis_11_2_cont.pdf10. *BulatovaNShchipanovNSearleJB (2007) The Seliger – Moscow hybrid zone between chromosome races of common shrews – an initial description.Russian Journal of Theriology6: 111-11611. *BulatovaNSearleJBBystrakovaNNadjafovaRShchipanovNOrlovV (2000) The diversity of chromosome races in *Sorex araneus* from European Russia.Acta Theriologica45: 33-4612.FrismanLVBorodinPMFrismanEY (2010) The evolutionary dynamics of hybrid zones of mammals.Regional Problems13: 56-61
[In Russian]13.Grigor’evaOOShestakAGPotapovSGBorisovYMIrkhinSYKorablevNPOrlovVN (2011) The microsatellite polymorphism and gene flow in the contact zone of four common shrew (*Sorex araneus* L., Mammalia) chromosome races.Izvestiya Academii Nauk5: 501–510
[English translation: Biology Bulletin 38: 425–433.]2211741614.Grigor’evaOOShestakAGSychevaVBPotapovSGBorisovYMOrlovVN (2011) Isolation effect of narrow hybrid zone of *Sorex araneus* chromosome races.Doklady Academii Nauk436: 830–833
[English translation: Doklady Biochemistry and Biophysics 436: 41–43.]10.1134/S16076729110101332136990215. *HornABassetPYannicGBanaszekABorodinPMBulatovaNSJadwiszczakKJonesRMPolyakovAVRatkiewiczMSearleJBShchipanovNAZimaJHausserJ (2012) Chromosomal rearrangements do not seem to affect the gene flow in hybrid zones between karyotypic races of the common shrew (*Sorex araneus*).Evolution66: 882-889.10.1111/j.1558-5646.2011.01478.x2238044610.1111/j.1558-5646.2011.01478.x16.KaramyshevaTVBelonogovaNMRodionovaMIRubtsovNBPolyakovAVSearleJBBorodinPM (2007) Temporal and spatial distribution of Rad51 protein in spermatocytes of the common shrew *Sorex araneus* L. (Soricidae, Eulipotyphla).Russian Journal of Theriology6: 15-1917.MatveevskySNPavlovaSVAcaevaMMKolomietsOL (2012) Synaptonemal complex analysis of interracial hybrids between the Moscow and Neroosa chromosomal races of the common shrew *Sorex araneus* showing regular formation of a complex meiotic configuration (ring-of-four).Comparative Cytogenetics6: 301-314.10.3897/CompCytogen.v6i3.37012426067010.3897/CompCytogen.v6i3.3701PMC383380518.OrlovVNBorisovYM (2007) Chromosome races of the common shrew *Sorex araneus* Linnaeus, 1758 (Mammalia: Insectivora) from the south part of Valdai Heights (Russia).Comparative Cytogenetics1: 101-10619. *OrlovVNBorisovYMCherepanovaEVGrigor’evaOOShestakAGSychevaVB (2012) Narrow hybrid zone between Moscow and Western Dvina chromosomal races and specific features of population isolation in common shrew *Sorex araneus* (Mammalia).Genetika48: 80–88
[English translation: Russian Journal of Genetics 48: 70–78.]10.1134/S10227954120101522256785720.OrlovVNBorisovYMCherepanovaEVMilishnikovAN (2013) Assortative mating in the hybrid zones of the common shrew (*Sorex araneus*, Mammalia) chromosome race West Dvina.Doklady Academii Nauk451: 110–113
[English translation: Doklady Biological Sciences 451: 217–220.]10.1134/S00124966130400172397546021.OrlovVNBorisovYMIrkhinSYKovalevaAA (2010) Characteristics of the contact zone of three chromosome races of the common shrew *Sorex araneus* L. (Mammalia) as indices of interpopulation competition.Ekologiya6: 459–463
[English translation: Russian Journal of Ecology 41: 519–523.]22. *OrlovVNKozlovskyAIOkulovaNMBalakirevAE (2007) Postglacial recolonisation of European Russia by the common shrew *Sorex araneus*.Russian Journal of Theriology6: 97-10423.OrlovVNSychevaVBCherepanovaEVBorisovYM (2013) Craniometric differences between karyotypic races of the common shrew *Sorex araneus* (Mammalia) as a result of limited hybridization.Genetika49: 479–490
[English translation: Russian Journal of Genetics 49: 417-427. doi: 10.7868/S0016675813040103]2386662510.7868/s001667581304010324. *PavlovaSV (2013) Cytogenetic analysis of a hybrid zone between the Moscow and Neroosa chromosomal races of the common shrew (*Sorex araneus*) differing by a single WART-like chromosome rearrangement.Tsitologiya55: 271-2742387546425.PavlovaSVBulatovaNSh (2010) Identification of a novel WART-like chromosome rearrangement in complex heterozygotes in an interracial hybrid zone of the common shrew *Sorex araneus* L.Genetika46: 1269–1271
[English translation: Russian Journal of Genetics 46: 1125-1126. doi: 10.1134/S1022795410090292]2106163226.PavlovaSVBulatovaNShShchipanovNA (2007) Cytogenetic control of a hybrid zone between two *Sorex araneus* chromosome races before breeding season.Genetika43: 1619–1626
[English translation: Russian Journal of Genetics 43: 1357–1363.]1859268827.PavlovaSVKolomietsOLBulatovaNShSearleJB (2008) Demonstration of a WART in a hybrid zone of the common shrew (*Sorex araneus* Linnaeus, 1758).Comparative Cytogenetics2: 115–120
www.zin.ru/journals/compcyt28.PavlovaSVBystrakovaNVBulatovaNSNadjafovaRSPolyakovAV (2006) Materials for cadastre of the chromosome races of the common shrew *Sorex araneus* L.Biogeography13: 42-59 [In Russian]29. *PollyPDPolyakovAVIlyashenkoVBOnischenkoSSWhiteTAShchipanovNABulatovaNSPavlovaSVBorodinPMSearleJB (2013) Phenotypic variation across chromosomal hybrid zones of the common shrew (*Sorex araneus*) indicates reduced gene flow.PLoS ONE8(7): .10.1371/journal.pone.006745510.1371/journal.pone.0067455PMC37079022387442030.PolyakovAV (2008) Hybrid zones between the chromosome races of the common shrew in West Siberia.Siberian Journal of Ecology15: 773-777
[In Russian]31. *PolyakovAVBorodinPMWhiteTAJonesRMSearleJB (2011) Natural hybridization between extremely divergent chromosomal races of the common shrew (*Sorex araneus*, Soricidae, Soricomorpha): Hybrid zone in Siberia.Journal of Evolutionary Biology24: 1393-1402.10.1111/j.1420-9101.2011.02266.x2150711410.1111/j.1420-9101.2011.02266.x32.PolyakovAVIlyashenkoVBOnischenkoSSPanovVBorodinP (2009) AFLP diversity between the Novosibirsk and Tomsk chromosome races of the common shrew (*Sorex araneus*).Comparative Cytogenetics3: 85-89.10.3897/compcytogen.v3i2.1433.PolyakovAVLadyginaTYuBochkarevMNRodionovaMIBorodinPMPanovVV (2001) Chromosomal evolution of the common shrew *Sorex araneus* L. from the Southern Urals and Siberia in the postglacial period.Genetika37: 448–455
[English translation: Russian Journal of Genetics 37: 351–357.]1142111734.PolyakovAVOnischenkoSSIliashenkoVBSearleJBBorodinPM (2002) Morphometric difference between the Novosibirsk and Tomsk chromosome races of the common shrew (*Sorex araneus*) in a zone of parapatry.Acta Theriologica47: 381-387.10.1007/BF0319246435.PolyakovAVVolobouevVTAniskinVMZimaJSearleJBBorodinPM (2003) Altitudinal partitioning of two chromosome races of the common shrew (*Sorex araneus*) in West Siberia.Mammalia67: 201-207.10.1515/mamm.2003.67.2.20136.PolyakovAVVolobouevVTBorodinPMSearleJB (1996) Karyotypic races of the common shrew (*Sorex araneus*) with exceptionally large ranges: the Novosibirsk and Tomsk races of Siberia.Hereditas125: 109-115.10.1111/j.1601-5223.1996.00109.x37.PolyakovAVZimaJBanaszekASearleJBBorodinPM (2000) New chromosome races of the common shrew *Sorex araneus* from eastern Siberia.Acta Theriologica45, Supplement 1: 11–1738. *SearleJBWójcikJM (1998) Chromosomal evolution: the case of *Sorex araneus*. In: WójcikJMWolsanM (Eds) Evolution of shrews.Mammal Research Institute, Polish Academy of Sciences, Białowieža, 219-26839.ShchipanovNABulatovaNShDemidovaTBBobretsovAV (2008) Chromosomal races of the common shrew (*Sorex araneus* L.) inhabiting northeastern European Russia: do physical obstacles restrict their distribution?Doklady Academii Nauk422: 714–717
[English translation: Doklady Biological Sciences 422: 348–351.]10.1134/s00124966080502191902469240. *ShchipanovNABulatovaNShPavlovaSVShchipanovAN (2009) The common shrew (*Sorex araneus*) as a model species in ecological and evolutionary studies.Zoologicheskii Zhurnal88: 975-989
[In Russian]41.ShchipanovNABulatovaNShPavlovaSV (2008) Distribution of two chromosome races of the common shrew (*Sorex araneus* L.) in the hybrid zone: can a change of the dispersal mode maintain independent gene frequencies?Genetika44: 734–745
[English translation: Russian Journal of Genetics 44: 635–645.]1872738342.ShchipanovNAPavlovaSV (2007) Hybridization of the common shrew (*Sorex araneus* L.) chromosomal races Moscow and Seliger: The probability of crossing and survival of hybrids.Doklady Academii Nauk of Russia417: 847–849
[English translation: Doklady Biological Sciences 417: 487–489.]10.1134/s00124966070602211827449943. *ShchipanovNAPavlovaSV (2013) Contact zones and ranges of chromosomal races of the common shrew, *Sorex araneus*, in northeastern European Russia.Folia Zoologica62: 24-35

## Index

Kirillov (Kr)
  gm, hi, kq, no, pr
  - Manturovo (F1: gm/mn/no/go, hi, kq, pr; RIV): 22, 43
  - Petchora (F1: gm/gi/hi/hn/no/mo, kq, pr; RVI): 39, 43
Manturovo (Ma)
  go, hi, kq, mn, pr
  - Kirillov (F1: gm/mn/no/go, hi, kq, pr; RIV): 22, 43
  - Petchora (F1: gi/hi/hn/mn/mo/go, kq, pr; RVI): 43
  - Sok (F1: go, kq, hi/ip/pr/mr/mn/hn; RVI): 43
Moscow (Mo)
  gm, hi, kr, no, pq
  - Neroosa (F1: gm/go/no/mn, hi, kr, pq; RIV): 17, 24
  - Seliger (F1: g/gm/mq/pq/pr/kr/ik/hi/hn/no/o; CXI): 1, 6, 8, 10, 15, 21, 23, 25, 26, 27, 29, 40, 41, 42
  - West Dvina (F1: gm, hi/ip/pq/qr/kr/hk, no; RVI): 6, 18, 20, 21, 23, 40
Neroosa (Ne)
  go, hi, kr, mn, pq
  - Moscow (F1: gm/go/no/mn, hi, kr, pq; RIV): 17, 24
Novosibirsk (No)
  go, hn, ik, mp, qr
  - Tomsk (F1: o/go/gk/ik/hi/hn/mn/mp/p, qr; CIX): 1, 11, 15, 16, 28, 29, 30, 31, 32, 34, 35, 36, 38
  - Serov (F1: go, hn, ik/ip/mp/km, qr; RIV): 28, 33
Petchora (Pt)
  gi, hn, kq, mo, pr
  - Kirillov (F1: gi/hi/hn/no/mo/gm, kq, pr; RVI): 39, 43
  - Serov (F1: gi/go/mo/km/kq/qr/pr/ip, hn; RVIII): 43
  - Sok (F1: gi/go/mo/mr/pr/ip, hn, kq; RVI): 43
Seliger (Sl)
  g, hn, ik, mq, o, pr
  - Moscow (F1: g/gm/mq/pq/pr/kr/ik/hi/hn/no/o; CXI): 2, 6, 8, 10, 15, 21, 23, 25, 26, 27, 29, 40, 41, 42
  - West Dvina (F1: g/gm/mq/qr/pr/ip/ik/hk/hn/no/o; CXI): 20
Serov (Se)
  go, hn, ip, km, qr
  - Novosibirsk (F1: go, hn, ik/ip/mp/km, qr; RIV): 28, 33
  - Petchora (F1: gi/go/mo/km/kq/qr/pr/ip, hn; RVIII): 43
  - Sok (F1: go, hn, ip, km/mr/qr/kq; RIV): 43
  - Yuryuzan (F1: go, hn, ip, km/mq/qr/kr; RIV): 40, 43
Sok (So)
  go, hn, ip, kq, mr
  - Manturovo (F1: go, kq, hi/ip/pr/mr/mn/hn; RVI): 43
  - Petchora (F1: gi/go/mo/mr/pr/ip, hn, kq; RVI): 43
  - Serov (F1: go, hn, ip, km/mr/qr/kq; RIV): 43
Strelka (Sr)
  go, hi, k, m, n, p, q, r
  - Tomsk (F1: k/gk/go/o, hi, q/r, m, n, p; CIV): 28, 37
Tomsk (To)
  gk, hi, mn, o, p, qr
  - Novosibirsk (F1: o/go/gk/ik/hi/hn/mn/mp/p, qr; CIX): 1, 11, 15, 16, 28, 29, 30, 31, 32, 34, 35, 36, 38
  - Strelka (F1: k/gk/go/o, hi, q/r, m, n, p; CIV): 28, 37
West Dvina (Wd)
  gm, hk, ip, no, qr
  - Moscow (F1: gm, hi/ip/pq/qr/kr/hk, no; RVI): 6, 19, 20, 21, 23, 40
  - Seliger (F1: g/gm/mq/qr/pr/ip/ik/hk/hn/no/o; CXI): 20
Yuryuzan (Yu)
  go, hn, ip, kr, mq
  - Serov (F1: go, hn, ip, km/mq/qr/kr; RIV): 40, 43

**Figure 1.**
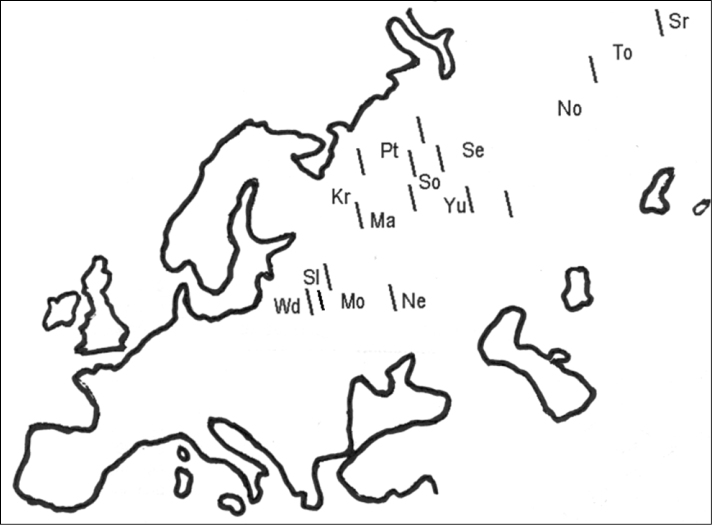
Schematic view of geographic distribution (slash) of hybrid zones between chromosome races of *Sorex araneus* in Russia. Standard abbreviations are used for the racial names (see Index).
